# Bis(acetone 4-phenyl­thio­semi­carbazonato-κ^2^ *N* ^1^,*S*)zinc(II)

**DOI:** 10.1107/S1600536809028244

**Published:** 2009-07-22

**Authors:** Kong Wai Tan, Chew Hee Ng, Mohd Jamil Maah, Seik Weng Ng

**Affiliations:** a Department of Chemistry, University of Malaya, 50603 Kuala Lumpur, Malaysia; b Faculty of Engineering and Science, Universiti Tunku Abdul Rahman, 53300 Kuala Lumpur, Malaysia

## Abstract

The Zn^II^ atom in the title compound, [Zn(C_10_H_12_N_3_S)_2_], is *N*,*S*-chelated by the deprotonated Schiff base in a tetra­hedral environment. The metal atom lies on a twofold rotation axis that relates one anion to the other. The amino H atom forms an intermolecular N—H⋯π inter­action to an aromatic ring.

## Related literature

For the two modifications of acetone 4-phenyl­thio­semicarbazone, see: Jian *et al.* (2005 ▶); Venkatraman *et al.* (2005 ▶).
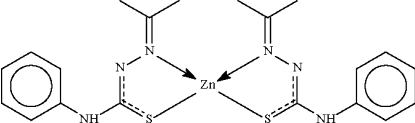


## Experimental

### 

#### Crystal data

[Zn(C_10_H_12_N_3_S)_2_]
*M* *_r_* = 477.94
Monoclinic, 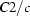

*a* = 23.5203 (5) Å
*b* = 7.2938 (2) Å
*c* = 15.1134 (5) Å
β = 122.761 (2)°
*V* = 2180.3 (1) Å^3^
*Z* = 4
Mo *K*α radiation
μ = 1.34 mm^−1^
*T* = 153 K
0.30 × 0.20 × 0.05 mm

#### Data collection

Bruker SMART APEX diffractometer
Absorption correction: multi-scan (*SADABS*; Sheldrick, 1996 ▶) *T*_min_ = 0.690, *T*_max_ = 0.936
6983 measured reflections
2488 independent reflections
1896 reflections with *I* > 2σ(*I*)
*R*_int_ = 0.031

#### Refinement

*R*[*F* ^2^ > 2σ(*F* ^2^)] = 0.039
*wR*(*F* ^2^) = 0.103
*S* = 1.10
2488 reflections
135 parameters
H-atom parameters constrained
Δρ_max_ = 0.40 e Å^−3^
Δρ_min_ = −0.45 e Å^−3^

### 

Data collection: *APEX2* (Bruker, 2008 ▶); cell refinement: *SAINT* (Bruker, 2008 ▶); data reduction: *SAINT*; program(s) used to solve structure: *SHELXS97* (Sheldrick, 2008 ▶); program(s) used to refine structure: *SHELXL97* (Sheldrick, 2008 ▶); molecular graphics: *X-SEED* (Barbour, 2001 ▶); software used to prepare material for publication: *publCIF* (Westrip, 2009 ▶).

## Supplementary Material

Crystal structure: contains datablocks I, global. DOI: 10.1107/S1600536809028244/bt5011sup1.cif

Structure factors: contains datablocks I. DOI: 10.1107/S1600536809028244/bt5011Isup2.hkl

Additional supplementary materials:  crystallographic information; 3D view; checkCIF report

## Figures and Tables

**Table 1:** Hydrogen-bond geometry (Å, °)

*D*—H⋯*A*	*D*—H	H⋯*A*	*D*⋯*A*	*D*—H⋯*A*
N3—H3⋯*Cg*^i^	0.86	2.86	3.671 (3)	157

Symmetry code: (i) 
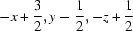
. *Cg* is the centroid of the aromatic ring.

**Fig. 1.**
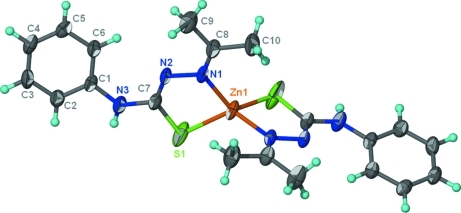
Anisotropic displacement ellipsoid (Barbour, 2001) plot of Zn(C10H12N3S)2 at the 70% probability level. Hydrogen atoms are drawn as spheres of arbitrary radii.

## supplementary crystallographic information

### Experimental

Zinc acetate monohydrate (0.22 g, 1 mmol) and 2,4-dihydroxybenzaldehyde 4-phenylthiosemicarbazone (0.29 g, 1 mmol) were heated in ethanol (50 ml) to form [1-(4-hydroxy-2-oxidobenzylidene)-4-phenylthiosemicarbazonato]zinc. The product was recrystallized from acetone, cleaved part of the anion.

### Refinement

Carbon-bound H-atoms were placed in calculated positions (C—H 0.95–0.98 Å) and were included in the refinement in the riding model approximation, with *U*(H) set to 1.2–1.5*U*_eq_(C). The amino H-atom was similarly treated (N–H 0.88 Å).

### Crystal data

**Table:**

[Zn(C_10_H_12_N_3_S)_2_]	*F*(000) = 992
*M**_r_* = 477.94	*D*_x_ = 1.456 Mg m^−^^3^
Monoclinic, *C*2/*c*	Mo *K*α radiation, λ = 0.71073 Å
Hall symbol: -C 2yc	Cell parameters from 2390 reflections
*a* = 23.5203 (5) Å	θ = 3.8–28.0°
*b* = 7.2938 (2) Å	µ = 1.34 mm^−^^1^
*c* = 15.1134 (5) Å	*T* = 153 K
β = 122.761 (2)°	Wedge, brown
*V* = 2180.3 (1) Å^3^	0.30 × 0.20 × 0.05 mm
*Z* = 4	

### Data collection

**Table:**

Bruker SMART APEX diffractometer	2488 independent reflections
Radiation source: fine-focus sealed tube	1896 reflections with *I* > 2σ(*I*)
graphite	*R*_int_ = 0.031
ω scans	θ_max_ = 27.5°, θ_min_ = 2.1°
Absorption correction: multi-scan (*SADABS*; Sheldrick, 1996)	*h* = −30→30
*T*_min_ = 0.690, *T*_max_ = 0.936	*k* = −9→9
6983 measured reflections	*l* = −19→19

### Refinement

**Table:**

Refinement on *F*^2^	Primary atom site location: structure-invariant direct methods
Least-squares matrix: full	Secondary atom site location: difference Fourier map
*R*[*F*^2^ > 2σ(*F*^2^)] = 0.039	Hydrogen site location: inferred from neighbouring sites
*wR*(*F*^2^) = 0.103	H-atom parameters constrained
*S* = 1.10	*w* = 1/[σ^2^(*F*_o_^2^) + (0.0387*P*)^2^ + 4.48*P*] where *P* = (*F*_o_^2^ + 2*F*_c_^2^)/3
2488 reflections	(Δ/σ)_max_ = 0.001
135 parameters	Δρ_max_ = 0.40 e Å^−^^3^
0 restraints	Δρ_min_ = −0.45 e Å^−^^3^

### Fractional atomic coordinates and isotropic or equivalent isotropic displacement parameters (Å^2^)

**Table:**

	*x*	*y*	*z*	*U*_iso_*/*U*_eq_	
Zn1	0.5000	0.30141 (7)	0.2500	0.03064 (17)	
S1	0.55393 (4)	0.14771 (13)	0.18550 (8)	0.0501 (3)	
N1	0.58756 (10)	0.4440 (3)	0.34189 (19)	0.0245 (5)	
N2	0.64335 (10)	0.3888 (3)	0.33788 (19)	0.0253 (5)	
N3	0.68276 (11)	0.1943 (4)	0.2634 (2)	0.0336 (6)	
H3	0.6712	0.1130	0.2154	0.040*	
C1	0.75216 (12)	0.2404 (4)	0.3216 (2)	0.0255 (6)	
C2	0.78857 (13)	0.1872 (4)	0.2775 (2)	0.0296 (6)	
H2	0.7662	0.1268	0.2113	0.035*	
C3	0.85719 (14)	0.2225 (4)	0.3300 (3)	0.0348 (7)	
H3A	0.8816	0.1866	0.2993	0.042*	
C4	0.89046 (13)	0.3090 (4)	0.4263 (3)	0.0332 (7)	
H4	0.9376	0.3330	0.4620	0.040*	
C5	0.85427 (13)	0.3606 (4)	0.4703 (2)	0.0289 (6)	
H5	0.8770	0.4194	0.5370	0.035*	
C6	0.78518 (13)	0.3275 (4)	0.4181 (2)	0.0283 (6)	
H6	0.7608	0.3646	0.4486	0.034*	
C7	0.63093 (13)	0.2588 (4)	0.2715 (2)	0.0275 (6)	
C8	0.59964 (13)	0.5716 (4)	0.4081 (2)	0.0302 (7)	
C9	0.66790 (15)	0.6551 (5)	0.4773 (3)	0.0456 (9)	
H9A	0.7007	0.5590	0.5196	0.068*	
H9B	0.6663	0.7453	0.5241	0.068*	
H9C	0.6815	0.7159	0.4338	0.068*	
C10	0.54313 (16)	0.6384 (5)	0.4182 (3)	0.0451 (9)	
H10A	0.5039	0.5575	0.3784	0.068*	
H10B	0.5307	0.7633	0.3903	0.068*	
H10C	0.5578	0.6380	0.4925	0.068*	

### Atomic displacement parameters (Å^2^)

**Table:**

	*U*^11^	*U*^22^	*U*^33^	*U*^12^	*U*^13^	*U*^23^
Zn1	0.0144 (2)	0.0296 (3)	0.0436 (3)	0.000	0.0129 (2)	0.000
S1	0.0171 (3)	0.0539 (6)	0.0665 (7)	−0.0064 (3)	0.0143 (4)	−0.0345 (5)
N1	0.0156 (9)	0.0257 (12)	0.0320 (14)	0.0020 (9)	0.0128 (10)	0.0034 (11)
N2	0.0148 (10)	0.0287 (12)	0.0321 (14)	0.0015 (9)	0.0124 (10)	−0.0009 (11)
N3	0.0196 (11)	0.0373 (14)	0.0408 (16)	−0.0017 (10)	0.0143 (11)	−0.0158 (13)
C1	0.0168 (11)	0.0235 (14)	0.0328 (16)	0.0030 (10)	0.0113 (12)	0.0006 (12)
C2	0.0245 (13)	0.0303 (15)	0.0332 (17)	0.0041 (12)	0.0152 (13)	−0.0016 (14)
C3	0.0241 (14)	0.0429 (18)	0.0432 (19)	0.0065 (13)	0.0221 (14)	0.0041 (16)
C4	0.0189 (12)	0.0373 (17)	0.0398 (18)	0.0023 (12)	0.0136 (13)	0.0086 (15)
C5	0.0216 (13)	0.0264 (14)	0.0310 (17)	0.0007 (11)	0.0092 (12)	0.0040 (13)
C6	0.0219 (13)	0.0307 (16)	0.0328 (17)	0.0034 (11)	0.0152 (13)	0.0004 (13)
C7	0.0171 (12)	0.0299 (15)	0.0328 (17)	−0.0001 (11)	0.0116 (12)	−0.0030 (13)
C8	0.0228 (13)	0.0258 (15)	0.0408 (18)	0.0015 (11)	0.0165 (13)	−0.0037 (14)
C9	0.0297 (15)	0.0361 (19)	0.063 (2)	−0.0023 (13)	0.0197 (17)	−0.0213 (17)
C10	0.0321 (16)	0.046 (2)	0.062 (2)	0.0046 (14)	0.0284 (17)	−0.0102 (18)

### Geometric parameters (Å, °)

**Table:**

Zn1—N1	2.039 (2)	C3—C4	1.376 (5)
Zn1—N1^i^	2.039 (2)	C3—H3A	0.9500
Zn1—S1	2.2702 (8)	C4—C5	1.386 (4)
Zn1—S1^i^	2.2702 (8)	C4—H4	0.9500
S1—C7	1.754 (3)	C5—C6	1.390 (4)
N1—C8	1.281 (4)	C5—H5	0.9500
N1—N2	1.405 (3)	C6—H6	0.9500
N2—C7	1.294 (4)	C8—C9	1.492 (4)
N3—C7	1.372 (3)	C8—C10	1.499 (4)
N3—C1	1.413 (3)	C9—H9A	0.9800
N3—H3	0.8600	C9—H9B	0.9800
C1—C6	1.381 (4)	C9—H9C	0.9800
C1—C2	1.395 (4)	C10—H10A	0.9800
C2—C3	1.384 (4)	C10—H10B	0.9800
C2—H2	0.9500	C10—H10C	0.9800
			
N1—Zn1—N1^i^	118.66 (13)	C5—C4—H4	120.5
N1—Zn1—S1	87.20 (6)	C4—C5—C6	120.9 (3)
N1^i^—Zn1—S1	123.54 (7)	C4—C5—H5	119.6
N1—Zn1—S1^i^	123.54 (7)	C6—C5—H5	119.6
N1^i^—Zn1—S1^i^	87.20 (6)	C1—C6—C5	119.7 (3)
S1—Zn1—S1^i^	120.82 (6)	C1—C6—H6	120.1
C7—S1—Zn1	92.67 (9)	C5—C6—H6	120.1
C8—N1—N2	115.1 (2)	N2—C7—N3	118.8 (2)
C8—N1—Zn1	128.17 (17)	N2—C7—S1	128.47 (19)
N2—N1—Zn1	116.57 (17)	N3—C7—S1	112.8 (2)
C7—N2—N1	114.8 (2)	N1—C8—C9	123.0 (2)
C7—N3—C1	130.2 (3)	N1—C8—C10	118.8 (3)
C7—N3—H3	114.9	C9—C8—C10	118.2 (3)
C1—N3—H3	114.9	C8—C9—H9A	109.5
C6—C1—C2	119.5 (2)	C8—C9—H9B	109.5
C6—C1—N3	124.5 (2)	H9A—C9—H9B	109.5
C2—C1—N3	116.0 (3)	C8—C9—H9C	109.5
C3—C2—C1	120.1 (3)	H9A—C9—H9C	109.5
C3—C2—H2	120.0	H9B—C9—H9C	109.5
C1—C2—H2	120.0	C8—C10—H10A	109.5
C4—C3—C2	120.8 (3)	C8—C10—H10B	109.5
C4—C3—H3A	119.6	H10A—C10—H10B	109.5
C2—C3—H3A	119.6	C8—C10—H10C	109.5
C3—C4—C5	119.1 (2)	H10A—C10—H10C	109.5
C3—C4—H4	120.5	H10B—C10—H10C	109.5
			
N1—Zn1—S1—C7	4.02 (12)	C2—C3—C4—C5	0.0 (5)
N1^i^—Zn1—S1—C7	126.96 (13)	C3—C4—C5—C6	−0.6 (4)
S1^i^—Zn1—S1—C7	−123.93 (11)	C2—C1—C6—C5	−0.3 (4)
N1^i^—Zn1—N1—C8	52.6 (2)	N3—C1—C6—C5	177.8 (3)
S1—Zn1—N1—C8	179.7 (3)	C4—C5—C6—C1	0.7 (4)
S1^i^—Zn1—N1—C8	−54.6 (3)	N1—N2—C7—N3	−179.0 (2)
N1^i^—Zn1—N1—N2	−132.0 (2)	N1—N2—C7—S1	1.3 (4)
S1—Zn1—N1—N2	−4.85 (18)	C1—N3—C7—N2	3.8 (5)
S1^i^—Zn1—N1—N2	120.81 (17)	C1—N3—C7—S1	−176.5 (3)
C8—N1—N2—C7	179.4 (3)	Zn1—S1—C7—N2	−4.3 (3)
Zn1—N1—N2—C7	3.4 (3)	Zn1—S1—C7—N3	176.0 (2)
C7—N3—C1—C6	18.0 (5)	N2—N1—C8—C9	−0.7 (4)
C7—N3—C1—C2	−163.9 (3)	Zn1—N1—C8—C9	174.8 (2)
C6—C1—C2—C3	−0.3 (4)	N2—N1—C8—C10	−179.7 (3)
N3—C1—C2—C3	−178.5 (3)	Zn1—N1—C8—C10	−4.1 (4)
C1—C2—C3—C4	0.4 (5)		

Symmetry codes: (i) −*x*+1, *y*, −*z*+1/2.

### Hydrogen-bond geometry (Å, °)

**Table:**

*D*—H···*A*	*D*—H	H···*A*	*D*···*A*	*D*—H···*A*
N3—H3···Cg^ii^	0.86	2.86	3.671 (3)	157

Symmetry codes: (ii) −*x*+3/2, *y*−1/2, −*z*+1/2.
